# Genome-Wide Identification and Transcriptome Analysis of P450 Superfamily Genes in Flax (*Linum usitatissimum* L.)

**DOI:** 10.3390/ijms26083637

**Published:** 2025-04-11

**Authors:** Yang Wu, Rula Sa, Yingnan Mu, Yu Zhou, Zhiwei Li, Xixia Song, Lili Tang, Dandan Liu, Liuxi Yi

**Affiliations:** 1 College of Agriculture, Inner Mongolia Agricultural University, Hohhot 010018, China; wuyang@imau.edu.cn (Y.W.); nndlizhiwei@imau.edu.cn (Z.L.); 2 College of Grassland Science, Inner Mongolia Agricultural University, Hohhot 010018, China; nndsarula@imau.edu.cn; 3 Institute of Crop Science, Inner Mongolia Academy of Agricultural & Animal Husbandry Sciences, Hohhot 010031, China; muyingnan19900222@163.com (Y.M.); zhouyu0419@126.com (Y.Z.); 4 Institute of Industrial Crops of Heilongjiang Academy of Agricultural Sciences, Harbin 150086, China; jjzwyjs@163.com (X.S.); tanglili19861126@126.com (L.T.); ldd5020@163.com (D.L.)

**Keywords:** flax genome, cytochrome, RNA-seq, cyanogenic glycosides, gene family

## Abstract

Flax (*Linum usitatissimum* L.) seed is rich in α-linolenic acid, lignans, and fiber, which have potential health benefits. However, the potential toxicity of its cyanogenic glycosides limits its widespread use. The cytochrome P450 gene family is one of the largest gene families in plants and is involved in synthesizing phytohormones, secondary metabolites, and various defense compounds. Two P450 genes have been found to be important enzymes for the biosynthesis of cyanogenic glycosides in common sorghum (*Sorghum bicolor* (L.) Moench). However, the P450 gene family and its involvement in cyanogenic glycoside synthesis have been less studied in flax. In previous studies, we assembled a high-quality flax genome. In this study, a total of 412 P450 genes were identified in the flax genome, with molecular weights in the range of 7.42 kDa to 154.5 kDa and encoding amino acid lengths between 67 and 1378. These genes belonged to 48 families under eight clans and were distributed across 15 chromosomes. The number of introns varied from 0 to 14. Thirty-nine cis-acting elements were identified within 1500 bp upstream of the promoter, mainly related to the light response. There were 147 segmental duplications and 53 tandem duplication events among these P450 genes. Eleven genes potentially related to cyanogenic glycoside synthesis were identified by transcriptome analysis, and the RT-qPCR results verified the reliability of the transcriptome analysis. This study lays the foundation for the classification and functional study of the flax P450 gene family. The results will be useful for breeding new low-cyanogenic-glycoside flax varieties by genetic engineering.

## 1. Introduction

Flax is an ancient plant that was cultivated in southwest Asia during the Neolithic period and later domesticated in Near Eastern agriculture [1]. Currently, it is widely cultivated in Asian, North American, South American, and European countries [2]. It can be divided into three main categories: fiber flax, oil flax, and dual-use flax. Flaxseed contains high levels of unsaturated fatty acids and nearly 30 anticancer compounds, which have been shown to lower cholesterol and blood pressure and reduce the risk of blood clots [3]. However, flax contains a potentially toxic substance called cyanogenic glycosides [4,5].

Cyanogenic glycosides are widely found in plants and play a role in their primary metabolisms. When plants are injured, cyanogenic glycosides release free toxic HCN from the wounds and inhibit the activity of heavy metal-containing enzymes and respiration [6]. This substance is also toxic to humans, which limits the widespread cultivation and utilization of flax [7].

Cytochrome P450 monooxygenase is one of the largest gene super-families in plants. It is named for the spectral absorbance peak of 450 nm that is produced when it binds carbon monoxide [8]. The modern cytochrome P450 gene originated from an ancestral gene some 3.5 billion years ago [9] and was first discovered in 1958 in rat and pig microsomes [9]. The plant P450 gene was first identified in cotton (*Gossypium hirsutum* L.) [10]. The P450 gene belongs to the group of oxidoreductases, which participates in NADPH^−^ and O^2−^-dependent hydroxylation processes with the cofactor heme thiol salts [11]. Its products are involved in the oxidative metabolisms of various endogenous and exogenous compounds in organisms through epoxidation, sulfation, dehalogenation, and dealkylation [12]. It plays a role in the plant growth, development, and biosynthesis of secondary metabolites [13,14].

A previous study found that the products of two P450 genes, CYP79A1 and CYP71E1, are critical enzymes in synthesizing cyanogenic glycosides in common sorghum [15]. CYP17A1 catalyzes the conversion of Tyr to Z-*p*-hydroxyphenylacetaldehyde oxime, followed by the conversion of Z-*p*-hydroxyphenylacetaldehyde oxime to *p*-hydroxymandelonitrile catalyzed by CYP71E1. Finally, a soluble glycosyltransferase UDP-Glc *p*-hydroxymandelonitrile glycosyltransferase UGT85B1 converts *p*-hydroxymandelonitrile to cyanogenic glycosides dhurrin [16].

The synthesis of the two raw cyanogenic glycosides in flax, linamarin, and lotaustralin begins with Val and Ile, respectively. The first step is N-hydroxylation to 2-methyl-propanal oxime or 2-methylbutanal-oxime, respectively. Subsequent dehydration forms 2-methylpropionitrile or 2-methylbutylnitrile, respectively. Then, oxygenation forms acetone/butanon cyanohydrin, which is then glycosylated by UDPG-glucosyltransferase to form linamarin and lotaustralin [17].

The above studies did not explore which genes control the onset of transformation of these compounds. Based on the fact that the process of cyanogenic glycoside synthesis is similar in sorghum and flax, and two important P450 genes in sorghum are involved in cyanogenic glycoside synthesis. The question remains: are there P450 genes in flax that are also involved in cyanogenic glycoside synthesis?

In this study, based on our newly assembled high-quality flax genome, the flax P450 genes were identified at the genome-wide level, and a series analysis including physicochemical properties, phylogenetic tree, chromosomal localization, gene structure, subcellular localization, and protein–protein interactions was performed. In addition, the expression levels of the P450 gene were determined in two inbred lines of flax with different cyanogenic glycoside contents at different times. Some P450 genes that may be involved in the synthesis of cyanogenic glycosides in flax were screened out. This study provides a basis for the research and application of the P450 gene in flax, as well as the cultivation of low cyanogenic glycoside flax varieties.

## 2. Result

### 2.1. LuP450 Genes Identification and Chromosomal Localization

After structural domain confirmation by SMART and CDD database, a total of 412 LuP450 genes were identified in the flax genome (Table S1).

**Figure 1:**
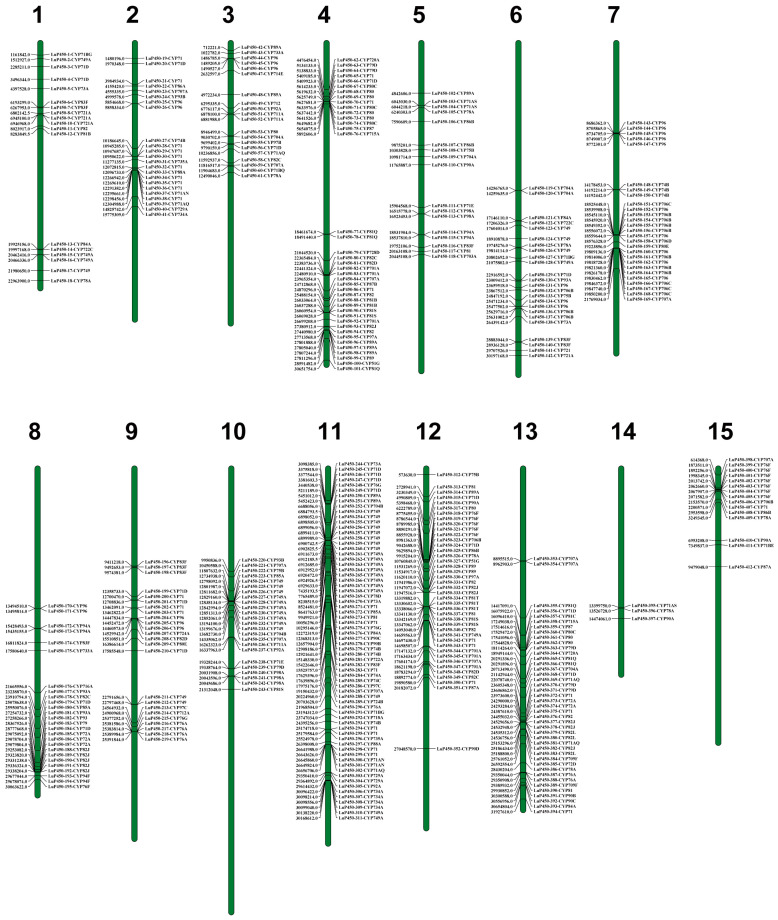
Distribution of LuP450 on flax chromosomes.

Chromosomal localization of the LuP450 genes showed that all LuP450 genes were unevenly distributed on 15 chromosomes. The highest number (68) was found on chromosome 11. The lowest number (three) was found on chromosome 14. Moreover, P450 is unevenly arranged in clusters on the chromosomes (Figure 1).

These genes were renamed as LuP450-1-CYP71BG to LuP450-412-CYP87A, according to their locations on the chromosomes and subfamilies, respectively. The length of the LuP450 protein sequences showed a wide variation, ranging from 67 to 1378 amino acids. Their molecular weights ranged from 7.42 kDa to 154.5 kDa, and Pi ranged from 4.14 to 11.26.

A-type and non-A-type P450 genes were unevenly distributed across different chromosomes. Among them, 33 A-type genes and eight non-A-type genes were found on chromosome 12, and the ratio of the number of A-type genes/non-A-type genes was 4.12. There were 32 A-type genes and eight non-A-type genes found on chromosome 4, and the ratio was 4, while the number of A-type genes on chromosomes 3, 10, and 11 were fewer than that of non-A-type genes, and the ratio was 0.82, 0.60, and 0.35, respectively.

### 2.2. Phylogenetic and Classification Relationship of LuP450 Genes

**Figure 2:**
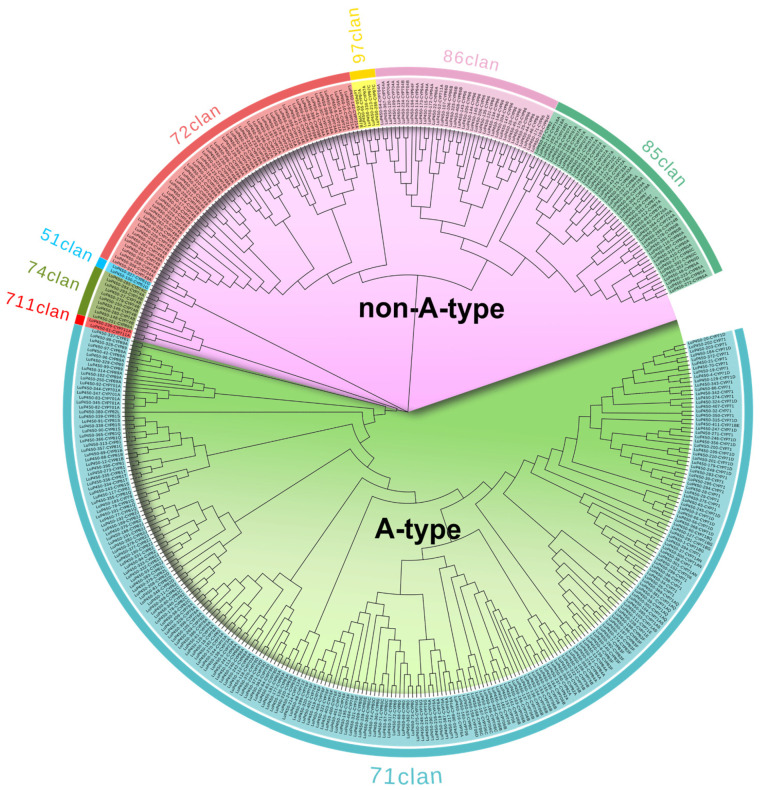
Phylogenetic tree of LuP450 genes.

To investigate the LuP450 gene’s phylogenetic relationships, we performed a phylogenetic analysis of 412 LuP450 genes using the maximum likelihood method. Of these genes, 248 (60.2%) belong to the A-type clade, and 164 (39.8%) belong to the non-A-type clade (Figure 2).

The A-type clade contains only one clan (71 clan), which was further divided into 19 families. The CYP71 family includes the largest number with 67 genes, and the CYP703 family has only one gene.

The non-A-type clade includes seven clans and was further divided into 29 families. Among them, CYP749 included the most genes at 40, while four families (CYP714, CYP716, CYP718, and CYP728) included only one gene.

To understand the location of the LuP450 gene action, subcellular localization prediction was performed using WoLF PSORT software (https://www.genscript.com/wolf-psort.html, accessed 29 March 2024). The results show that 63.11% (260/412) of the LuP450 protein was localized in chloroplasts, 18.45% (76/412) was localized in the cytoplasm, and 5.34% (22/412), and 5.10% (21/412) were localized in the plasma membrane and nucleus, respectively.

### 2.3. Conserved Motif and Gene Structure Analysis of LuP450 Genes

To further compare the LuP450 gene structures, we performed conserved domain and gene structure analyses. A total of 10 conserved motifs were identified through the MEME analysis (Figure S1).

The number of motifs of the protein sequences of the LuP450 genes varied from 1–24 (Table S2).

Among them, motif 2 (K-helix region), motif 1 (C-terminal heme-binding region), motif 3 (PXRX motif), and motif 6 (I-helix oxygen-binding region) appeared in 332,300,300 and 276 genes, respectively. Only 231 genes had all four typical domains. The distribution of motifs varies in different clans. Motif 4 was not identified in genes belonging to the 97 clan, motif 9 was not identified in genes belonging to the 51 clan, and motif 7 was found only in genes under the 71 clan. The results show that there is a certain degree of specificity in motifs among different clans and demonstrate the reliability of the phylogenetic tree construction.

To understand the LuP450 gene structure, we analyzed the introns–exons based on the gff files. The results show that the number of introns in the 412 LuP450 genes varied from 0 to 14 (Figure S1, Table S3). Among them, 104 genes had no introns, and 135 genes had only one intron. In addition, we found that exon–intron specificity may exist in some clans; for example, 72.6% of 71 clan genes have only 1–2 introns, while all 97 clan genes have more than seven introns.

### 2.4. Cis-Acting Elements Analysis of LuP450 Genes

Cis-acting elements play important roles in regulating gene expression. We analyzed cis-acting elements in the 1500 kb region upstream of the LuP450 gene promoter using the PlantCARE website.

A total of 8542 cis-acting elements were identified on 412 LuP450 genes, which were categorized into 29 classes, with 17 classes having more than 50 elements (Figure S1).

**Figure 3:**
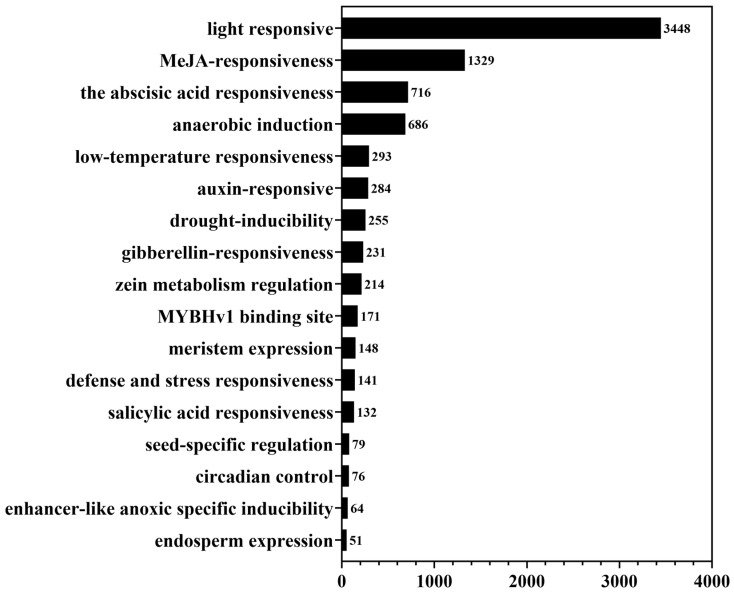
Cis-acting element distribution of the LuP450 genes.

The light response elements were the most abundant (3448 in total) and were identified on 99% (408/412) of LuP450 genes (Figure 3). There were at least two light response elements in each gene, with a maximum of 20 identified in the LuP450-21-CYP71.

Hormone-responsive elements were also abundantly present upstream of the LuP450 promoter, such as MeJA responsiveness, abscisic acid responsiveness, auxin responsiveness, and gibberellin responsiveness, with total numbers of 1329, 716, 284, and 231 elements respectively.

There is also a high content of biotic stress-response components, such as anaerobic induction, low-temperature responsiveness, drought-inducibility, and defense and stress responsiveness, with total numbers of 686, 293, 255, and 141, respectively.

In addition, some elements related to growth and development were also identified, such as zein metabolism regulation, meristem expression, seed-specific regulation, circadian control, and endosperm expression, and their numbers were 214, 148, 79, 76, and 51, respectively.

### 2.5. Intragenomic Covariance Analysis of the LuP450 Gene

To explore the evolution and expansion of the LuP450 gene within the flax genome, we performed fragment-duplication and tandem-duplication event analyses using BLASTP (2.2.26) and MCscanX software.

**Figure 4:**
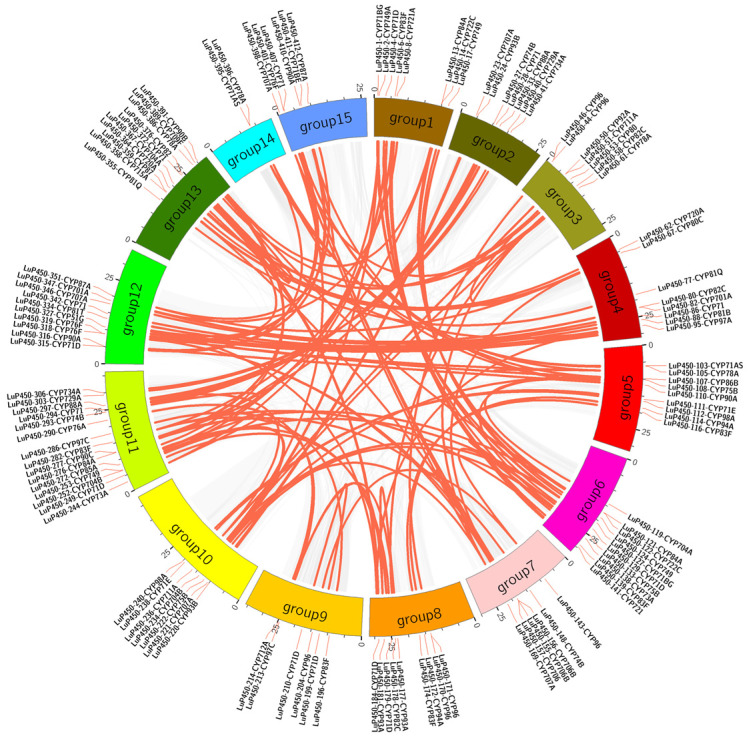
Intragenomic covariance analysis of the LuP450 genes. The gray lines in the background indicate other gene collinear blocks of flax, and the red lines indicate the syntenic P450 gene pairs.

There were 202 genes that formed 147 segmental duplication events through different combinations, of which 59 genes had multiple duplications (Figure 4, Table S4). These genes were distributed on 15 chromosomes. The maximum number of genes was 24 on chromosome 11, and the minimum number of genes was three on chromosome 14.

A total of 53 tandem duplications involving 97 genes were found (Table S5), of which seven tandem duplications were repeated three times (*LuP450-28-CYP71*+*LuP450-30-CYP71*, *LuP450-224-CYP749*+*LuP450-226-CYP749*, *LuP450-334-CYP81T*+*LuP450-336-CYP81T*, *LuP450-403-CYP76F*+*LuP450-405-CYP76F*, *LuP450-71-CYP80C*+*LuP450-73-CYP80*, *LuP450-96-CYP89A*+*LuP450-98-CYP89A*, and *LuP450-143-CYP96*+*LuP450-145-CYP96*) and one was repeated four times (*LuP450-162-CYP706*+*LuP450-165-CYP706*). The results suggest a complex co-collinearity among the LuP450 genes.

### 2.6. Analysis of P450 Gene Covariance Among Different Genomes

To investigate the selection pressure between segmental duplication and tandem duplication gene pairs, we performed Ka/Ks analysis on 201 of the gene pairs screened in the previous step. The results reveal that except for four gene pairs for which Ka/Ks was not calculated, all other gene pairs had Ka/Ks ratios of less than 1, indicating that the LuP450 gene was under purification selection pressure (Table S6).

**Figure 5:**
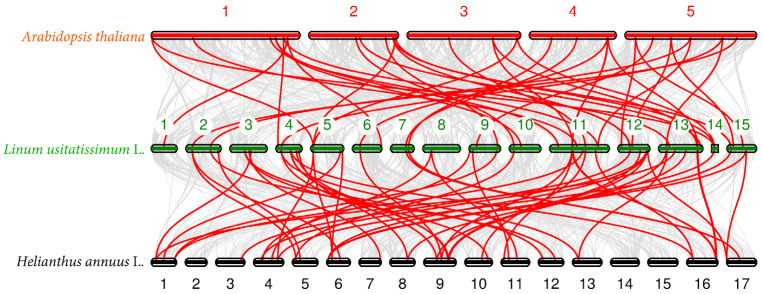
Synteny analysis of P450 genes among three genomes. The gray lines in the background indicate other gene collinear blocks of flax, and the red lines indicate the syntenic P450 gene pairs.

To understand the phylogenetic relationship of P450 genes in flax and other species, we performed a collinearity analysis with the flax genome, using Arabidopsis and sunflower genomes. The results show that a total of 40 pairs of colinear P450 genes were identified in Arabidopsis and flax, and a total of 58 pairs of colinear P450 genes were identified in sunflower and flax (Figure 5).

### 2.7. Expression Pattern Analysis of LuP450 Genes

**Figure 6:**
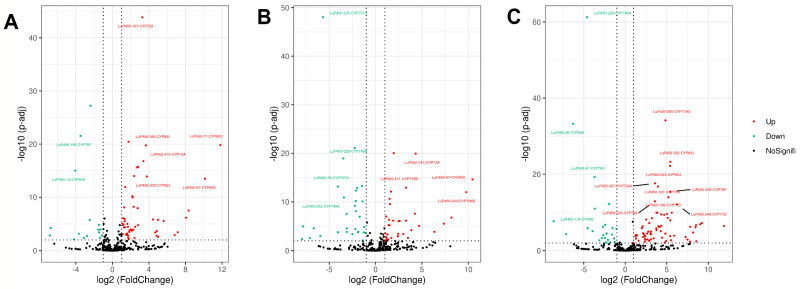
LuP450 differentially expressed gene volcano plot. (**A**) A15S vs. A224S. (**B**) A15M vs. A224M. (**C**) A15L vs. A224L. Each dot represents a differentially expressed gene, and genes with -log(padj) > 10 while abs(log2FoldChange) > 3 were labeled.

To investigate the expression of the LuP450 gene in A15 and A224, transcriptome analysis was carried out for three developmental periods (Figure S2). Differential expression analysis showed that there were 69 (53up/16down), 64 (34up/30down), and 94 (69up/25down) DEGs in A15S/A224S, A15M/A224M, and A15L/A224L, respectively (Figure 6).

**Figure 7:**
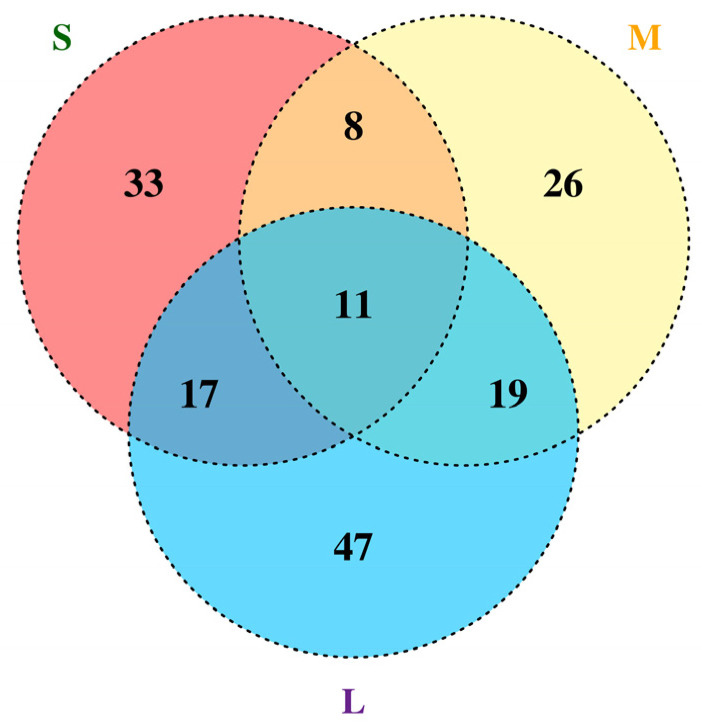
Venn diagram of LuP450 differentially expressed genes at three developmental periods.

Eleven genes (LuP450-383-CYP82L, LuP450-355-CYP81Q, LuP450-395-CYP71AS, LuP450-187-CYP72A, LuP450-357-CYP81C, LuP450-189-CYP82J, LuP450-389-CYP709F, LuP450-249-CYP71D, LuP450-411-CYP71BE, LuP450-71-CYP80C, and LuP450-67-CYP80C) were present in all three comparison groups simultaneously (Figure 7).

Nine of the genes belonged to the CYP71, CYP80, CYP81, and CYP824 subfamilies under the 71 clan, and two genes belonged to the CYP72 and CYP709 subfamilies under the 72 clan. All nine genes were upregulated for expression in A224, and the log_2_FoldChange for the *LuP450-411-CYP71BE* gene was greater than nine in all three comparison groups.

### 2.8. RT-qPCR Validation

**Figure 8:**
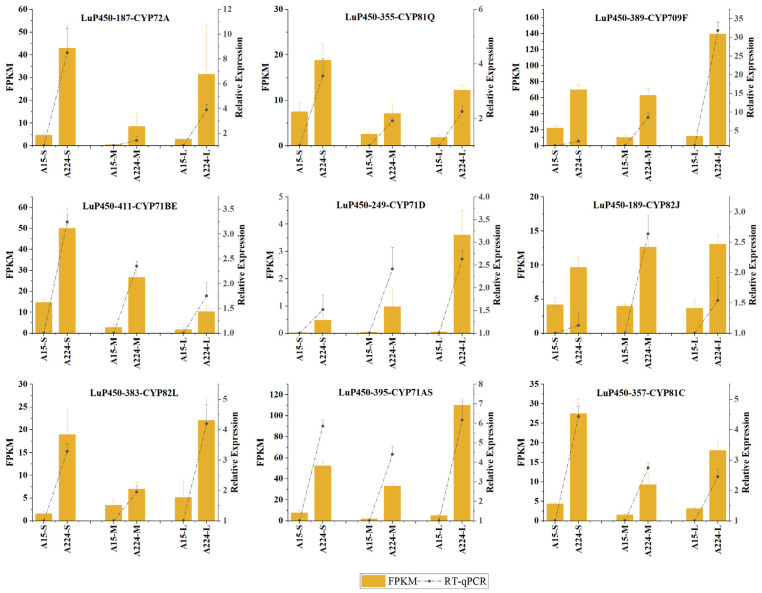
The relative expression of nine genes was analyzed by RT-qPCR, and the dotted line plots are drawn jointly with the FPKM value.

The RT-qPCR results showed a consistent trend in the relative expressions of all genes between FPKM, proving the reliability of the transcriptome analysis (Figure 8).

### 2.9. Protein–Protein Interaction Analysis

**Figure 9:**
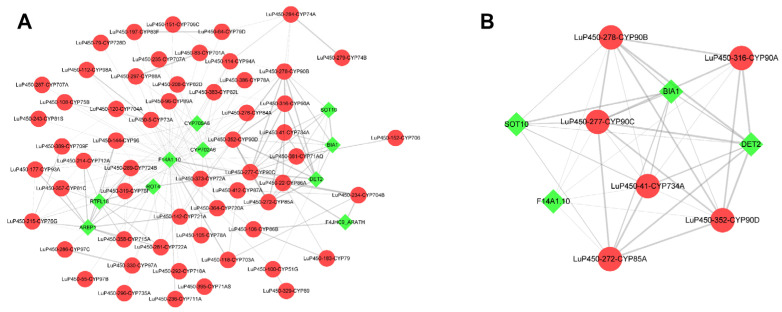
LuP450 protein interactions network map. Red dots represent flax P450 genes and green diamonds represent other genes. (**A**) All LuP450 protein interactions network map. (**B**) Core network map calculated by the MCODE plugin.

To study the interactions among the LuP450 proteins and screen hub genes, we performed a protein–protein interaction (PPI) analysis and constructed a PPI network. This network contained 68 nodes and 186 edges (Figure 9A). We further calculated the hub genes using the MCODE plugin and obtained a new network containing 10 nodes and 37 edges (Figure 9B) with six LuP450 genes (LuP450-41-CYP734A, LuP450-272-CYP85A, LuP450-277-CYP90C, LuP450-278-CYP90B, LuP450-316-CYP90A, and LuP450-352-CYP90D). All these genes belong to the 85 clan except the LuP450-41-CYP734A gene, which belongs to the 72 clan.

## 3. Discussion

The cytochrome P450 (CYP) superfamily includes a wide range of hemeproteins, and it is the largest enzyme–protein superfamily among living organisms [18]. Originating in prokaryotes, the P450 gene has since been demonstrated to be widely distributed in plants, animals, fungi, yeasts, and algae. The P450 gene in plants was first discovered in cotton [10] and is significantly more abundant in higher plants than in other organisms [19]. Currently, there are more than 300,000 gene sequences in the P450 gene database, of which more than 16,000 plant P450 genes have been named [20]. P450 genes make up roughly 1% of the total number of plant genes, with the proportion varying in different plants. There were 245 P450 genes found in *Arabidopsis thaliana* (L.) [21], 74 in *P. bretschneideri* [22], 223 in tomato (*Solanum lycopersicum* L.) [23], 351 in sorghum [24], 273 in tea plant (*Camellia sinensis*) [14], 376 in alfalfa (*Medicago sativa* L.) [25], 589 in peanuts (*Arachis hypogaea* L.) [26], 478 in pepper (*Capsicum annuum* L.), and 279 in cabbage (*Brassica oleracea*) [27]. This study identified 412 P450 genes in the flax genome, accounting for 1.25% (412/32785) of the total number of genes. Compared with a decade-old study [28], 78 more genes were identified, which will help us to better understand and utilize P450 genes for genetic breeding at the molecular level.

P450 genes were initially categorized into two types, the A-type clade and non-A-type clade, with A-type encoding mainly plant-specific secondary product metabolizing enzymes, while non-A-type mainly function in lipid or hormone metabolisms [8]. However, the classification of the P450 gene into only two types is clearly insufficient for such a large gene family, and more detailed classification and nomenclature is required. Based on the phylogenetic tree clustering results, the nomenclature committee subdivided the P450 gene into 11 clans, including one clan (71 clan) under the A-type clade and the other 10 clans under the non-A-type clade.

The P450 genes under each clan were named according to the sequence consistency, and their names consist of “CYP + number + letter.” If two genes have more than 40% amino acid sequence identity, they are categorized into one family and use the same number. Furthermore, if the amino acid sequence identity is more than 55%, the last letter is also the same, and they are categorized into the same subfamily [29].

Based on this nomenclature rule, 147 families have been identified in 11 clans of plants (CYP71-CYP99 and CYP701-CYP999), of which four clans contain multiple families (71 clan, 72 clan, 85 clan, and 86 clan) and seven clans contain only one family (51 clan, 74 clan, 97 clan, 710 clan, 711 clan, 727 clan, and 746 clan) [21].

Of the 412 LuP450 genes identified in this study, 248 A-type genes were categorized into 17 families under the 71 clan, and 164 non-A-type genes were categorized into 29 families under seven clans. Forty CYP749 subfamily genes were identified in this study, whereas no genes belonging to this subfamily were identified in previous studies [28]. In addition, 10 more genes were identified in the CYP81 and CYP83 families, respectively. This study identified more P450 genes, probably because we used a more comprehensive new reference genome annotation, and we used two identification strategies.

The P450 gene has four highly conserved structural domains: 1. the PXRX motif (“FXPERF” for A-type and “FXPXRX” for non-A-type); 2. the I-helix oxygen-binding domain (“AGXDT” for A-type and “AGX[D/E]T” for non-A-type); 3. the C-terminal heme-binding region (“PFGXGRRXCXG” for A-type and “XFXXGXRXCXG” for non-A-type); 4. the K-helix region (EXXR), which is consistent with both A-type and non-A-type [27,30,31].

This study found that only 56% of the LuP450 genes have all four typical structural domains simultaneously, suggesting that some of the structural domains may not be essential for accomplishing specific functions.

The P450 superfamily found in plants has a thioredoxin catalytic center, usually attached to the endoplasmic reticulum (ER) [32]. In this study, most of the P450 genes were found to be localized to chloroplasts and followed by the ER. This result is consistent with the study of the P450 gene family in tea plant [14] and *Medicago sativa* L. [25]

Gene structure analysis revealed that the flax P450 gene structure is relatively simple, with 58% containing only 1–2 exons, a result similar to that reported in pear (*Pyrus* spp.) [33]. A study in Medicago truncatula found that intron–exon ratios were specific across families, e.g., most CYP71 family members contained only one zero-phase intron, whereas CYP97B13, CYP96J18, and CYP97A10 contained more than 10 introns [34]. This study found distributional specificity in individual clans, such as most 71 clan members contain less than one intron, while 97 clan members contain more introns and have complex structures, suggesting that members of the same clan may have similar structures and functions.

Cis-acting elements are critical units of transcriptional regulation and are involved in the regulation of molecular networks [35]. This study found that the cis-acting elements of the LuP450 genes are diverse, with the highest content of light-responsive elements, and the number of elements related to hormone response, abiotic stress response, and growth and development was also higher, indicating the diversity of LuP450 gene functions. This result is also supported by the studies of foxtail millet (*Setaria italica* L.) [36] and grapevine (*Vitis vinifera* L.) [11].

Gene duplication is a key factor driving the evolution of species, through which gene families are expanded and species acquire new functions to accommodate environments [37]. The main forms are tandem and segmental duplication, the former associated with the origin of polyploidy and the latter with rapid adaptation to unfavorable environmental pressures [38]. The diversity of the P450 gene superfamily was introduced through an extensive process of gene duplication [8].

In this study, 147 segmental duplication events and 53 tandem duplication events were observed, with a ratio of 2.77 (147/53). This ratio is similar to that of peanut (*Arachis hypogaea* L.) (2.63), suggesting that fragment duplication is the main form of gene duplication [26]. In other species, such as tea plant (*Camellia sinensis*), pepper (*Capsicum annuum* L.), and foxtail millet (*Setaria italica* L.), tandem duplication events are the dominant form of gene duplication, and the ratios between segmental duplication and tandem duplication events are 0.75, 0.22, and 0.34, respectively [14,36,39].

Ka/Ks represents the ratio between the rate of nonsynonymous substitutions (Ka) and the rate of synonymous substitutions (Ks) in two protein-coding genes. This ratio can indicate whether there is a selective pressure on this protein-coding gene [40].

Ka/Ks > 1 indicates that the gene is under positive selection, Ka/Ks = 1 indicates neutral evolution, and Ka/Ks < 1 indicates purify selection [41]. In this study, all the paired genes’ Ka/Ks were found to be less than 1, indicating that the genes were subject to purification selection.

Cyanogenic glycosides belong to a group of antimicrobial compounds that protect plants from infection by providing defense against fungi, bacteria, and viruses. Two P450 family genes, CYP79 and CYP71, have been proven to be involved in the synthesis of raw cyanogenic glycosides in *Sorghum bicolor* (L.) Moench [15] and cassava (*Manihot esculenta*) [42]. In sorghum, it is involved in dhurrin biosynthesis. In cassava, CYP79 family genes catalyze the conversion of L-valine and L-isoleucine to the corresponding oximes, which are then catalyzed by CYP71 to form the corresponding cyanohydrin and finally glycosylated by UDP glucosyltransferases. Six homologs of the CYP79D1 and two CYP71E1 genes were identified in flax, which may be involved in the synthesis of cyanogenic glycosides [42].

In this study, two flax inbred lines with large differences in cyanogenic glycoside contents were selected and sampled at three developmental periods. Transcriptome analysis was utilized to find differentially expressed LuP450 genes, and 69, 64, and 94 differentially expressed LuP450 genes were identified in the three periods, respectively. There were 11 differentially expressed genes that were present in all three periods simultaneously, and they were more highly expressed in varieties with lower cyanogenic glycoside contents. The accuracy of transcriptome analysis was verified by RT-qPCR experiments. Six hub genes were obtained by protein interaction analysis. These genes may be involved in the synthesis of cyanogenic glycosides in flax and are important targets for our following functional validation.

The results of this study will help to reveal the regulatory mechanism of flax cyanogenic glycoside synthesis. Additionally, it will provide a basis for using genetic engineering to cultivate new flax varieties with low cyanogenic glycosides.

## 4. Materials and Methods

### 4.1. Identification Physiological Features of LuP450 Genes

The flax genome, protein sequence, and annotation files use our previously assembled method and can be downloaded from the Zenodo website (https://zenodo.org/records/7811972, accessed on 8 October 2023) [43].

The Hidden Markov Model file (PF00067) was downloaded from the Pfam database (http://pfam.xfam.org/, accessed on 2 February 2024). The HMMER (V3.1b2) software was used to identify P450 genes with default parameters.

In addition, we used a BLAST method for the complementary identification of the P450 gene. Briefly, 255 *Arabidopsis thaliana* P450 protein sequences were downloaded from the TAIR website. The BLAST databases were built using the makeblastdb command. Then, the flax protein sequences were used as query sequences for a local BLASTP comparison analysis with an E-value threshold of 1 × 10^−10^.

The putative P450 gene protein sequences were then submitted to the CDD, SMART, and PFAM databases for conserved domain confirmation, and the genes that did not have P450 typical structural domains were deleted in subsequent analyses.

The finalized P450 genes were renamed as “LuP450” and numbered according to the order they appeared on the chromosomes plus the subfamily they belong to. For example, the “*LuP450-22-CYP86A*” indicates the 22nd occurrence of the flax P450 gene belonging to the CYP86A subfamily. The molecular weight (Mw) and isoelectric point (pI) were determined using SeqStats and the pICalculator Bioperl module [44].

### 4.2. Phylogenetic Analysis of LuP450 Genes

All LuP450 protein sequences were aligned using the ClustalW algorithm in the MEGA 7 software with default parameters to clear up the evolutionary relationship. The phylogenetic tree was constructed using the maximum likelihood method (Jones–Taylor–Thornton model with 1000 bootstrap replicates). The phylogenetic tree file was then submitted to the Evolview V2 website (https://evolgenius.info//evolview-v2/#login, accessed 29 March 2024) [45] for further visualization and annotation.

### 4.3. Conserved Domain and Motif Analysis of LuP450 Genes

The LuP450 conserved motif analysis was conducted using the Multiple Em for Motif Elicitation website (MEME; https://meme-suite.org/tools/meme, accessed 29 March 2024). The total number of motifs searched was set to 10, with a minimum length of six amino acids and a maximum length of 100 amino acids.

### 4.4. Gene Structure, Chromosomal Localization, and Cis-Acting Elements Analysis of LuP450 Genes

A Perl script was used to obtain information about the exon, CDS, and UTR positions of the LuP450 genes. The physical location of the LuP450 gene was retrieved from the gff3 file, and the chromosome lengths were obtained using SAMtools software (1.15.1). The gene chromosomal localization was visualized using MapChart software (2.3.2). To identify the cis-acting elements of the LuP450 genes, 1500 kb of gene sequence upstream of the start codon (ATG) was extracted. The cis-acting elements were identified using the Plant Cis-Acting Regulatory Element (PlantCARE, https://bioinformatics.psb.ugent.be/webtools/plantcare/html/, accessed 21 February 2024) [46].

The “gene structure view feature” function of the TBtools software (2.142) [47] was used to present the phylogenetic tree, motif, gene structure, and cis-acting elements in a single figure.

### 4.5. Subcellular Localization Prediction of LuP450 Genes

The advanced online computational tool WoLF PSORT [48] (PSORT II, http://www.genscript.com/wolf-psort.html, accessed 17 May 2024) program was used to predict the LuP450 gene subcellular localization.

### 4.6. Intragenomic Covariance Analysis of LuP450 Genes

Genome-wide segmental analysis and tandem deduplication event analysis were conducted using MCscanX software with default parameters. These events were visualized using Circos software (0.69-8). To determine the selection pressure of LuP450 genes, the synonymous (Ka) and non-synonymous (Ks) substitution rates between LuP450 gene pairs were calculated using the Kaks Calculator with the YN method. The divergence times of the LuP450 genes from their ancestors was estimated using the following equation: T = Ks/2λ, and for dicotyledons, λ = 1.5 × 10^−8^ [49].

### 4.7. Intergenomic Covariance Analysis of the LuP450 Gene

The synteny comparison among *Linum usitatissimum* L., *Helianthus annuus* L., and *Arabidopsis thaliana* was performed using MCscan (https://github.com/tanghaibao/jcvi/wiki/MCscan-(Python-version), accessed 29 March 2024).

The CDS sequences and gff annotation files were downloaded from the Phytozome website. The chromosome-scale pairwise synteny search was performed using the javi.compara.catalog command. The macrosynteny were visualized using jcvi.graphics.karyotype command.

### 4.8. LuP450 Gene Expression Analysis

To investigate the expression pattern of LuP450 genes, we chose flax inbred line A15 with high cyanogenic glycoside content and A224 with low cyanogenic glycoside content for transcriptome analysis. Flax capsules were sampled on the day of flowering, on the 15th day after flowering, and on the 30th day after flowering, respectively.

Total RNA was extracted using the TransZol Up Plus RNA kit (Transgen, Beijing, China), followed by cDNA first-strand synthesis using the MightyScript First Strand cDNA Synthesis Master Mix (Tailing Reaction, Auckland, New Zealand).

High-quality reads were aligned to the reference genome using Hisat2 (2.2.1) software. Gene expression levels were expressed using fragments per kilobase of the exon model per million mapped fragments (FPKM) values. The DESeq2 Rpackage (1.40.2) was used for differentially expressed gene (DEG) analysis.

Three comparison combinations, A15S/A224S, A15M/A224M, and A15L/A224L, were set up to represent the expression of the LuP450 genes in the two materials.

The letters S, M, and L indicate the day of flowering, 15 days after flowering, and 30 days after flowering, respectively. The threshold for DEG was set as padj < 0.05 and abs(log2FoldChange) > 1.

To verify the accuracy of transcriptome analysis, 9 differentially expressed LuP450 genes were randomly selected for RT-qPCR validation. The experiments were performed on a FTC-3000TM Real-Time Quantitative Thermal Cycler (Funglyn Biotech, Toronto, ON, Canada) instrument. Gene-specific primers were designed using the free online primer design tool (https://www.sangon.com/newPrimerDesign, accessed 29 March 2024).

Three replicates were performed for each sample, and gene expression was calculated by 2^−△△Ct^ method, with EF1A-F gene as an endogenous control. The sequence information is shown in Table 1.

### 4.9. Protein–Protein Interaction Network Analysis of LuP450 Genes

To investigate the interactions between LuP450 proteins, we performed a protein–protein interaction (PPI) analysis. The homologs of the LuP450 genes in *Arabidopsis* were obtained using the OrthoVenn3 platform (https://orthovenn3.bioinfotoolkits.net/start/db, accessed 29 March 2024) [50]. Protein interaction networks were obtained using the STRING website (https://string-db.org, accessed 29 March 2024) [51]. The network was visualized and optimized using Cytoscape software (3.8.2) [52]. Then, we used the MCODE plugin [53] to screen for hub genes with node degreed cutoff = 5, cluster finding method = Haircut, score cutoff = 0.2, k-core = 2, and maximum depth = 100.

## 5. Conclusions

In this study, 412 LuP450 genes were identified on our assembled genome and were analyzed for physiological features, phylogenetic relationships, conserved domains, gene structures, cis-acting elements, fragments, and tandem duplication events, which help us to understand their classifications and properties. Transcriptome analysis was performed using two flax inbred lines with different raw cyanogenic glycoside contents at three time points, and 11 common DEGs were obtained (*LuP450-383-CYP82L*, *LuP450-355-CYP81Q*, *LuP450-395-CYP71AS*, *LuP450-187-CYP72A*, *LuP450-357-CYP81C*, *LuP450-189-CYP82J*, *LuP450-389-CYP709F*, *LuP450-249-CYP71D*, *LuP450-411-CYP71BE*, *LuP450-71-CYP80C*, and *LuP450-67-CYP80C*). Additionally, six hub genes were obtained using protein interaction analysis (*LuP450-41-CYP734A*, *LuP450-272-CYP85A*, *LuP450-277-CYP90C*, *LuP450-278-CYP90B*, *LuP450-316-CYP90A*, and *LuP450-352-CYP90D*). These genes will be made a focus for functional validation and used for low-cyanogenic glycoside flax breeding.

## Figures and Tables

**Table 1:** Primers for RT-qPCR validation analysis.

Gene ID	Forward Primer	Reverse Primer
EF1A-F	GCTGCCAACTTCACATCTCA	GATCGCCTGTCAATCTTGGT
LuP450-357-CYP81C	CACACACTCACCCAACGCTACG	CGACGGACGGCGAGGAGAG
LuP450-355-CYP81Q	GACATGGAAGAGGCGGATCAGTTC	GCCAAAGCCAACCCATTTCAAGAG
LuP450-187-CYP72A	TGATGATGCTGGCGAGTTTAACCC	TCCTCGGACCCCACCCAAATG
LuP450-411-CYP71BE	CGTCAAGTGTGATAGCCAGGTCAG	ACCTCCAGCCAACTCCAGACTG
LuP450-249-CYP71D	AGCGGCGAGGGAGGTGTTC	TCGGAGCGGTCGTAGGTGATG
LuP450-383-CYP82L	CTTCTGTCCAACCACCGCATCG	AACGCCAACTCCTCCAACAACTG
LuP450-189-CYP82J	CGTTCCACCACCAGTCATCCAC	AGAGGGTTAGGGCGAGAGGTTTC
LuP450-389-CYP709F	ACCCAGTATCTTCCTACGCCATCG	CCATAACCACCACCACCACCTTTG
LuP450-395-CYP71AS	GACAAAGGCAGGGTGACAGAAGAC	GAGAGGAGCGGGTGGGTGAAG

## Data Availability

Data will be made available upon request.

## Supplementary Materials

The following supporting information can be downloaded at https://www.mdpi.com/article/10.3390/ijms26083637/s1.
